# The Effect of Temperature on the Toxicity of Insecticides against *Musca domestica* L.: Implications for the Effective Management of Diarrhea

**DOI:** 10.1371/journal.pone.0095636

**Published:** 2014-04-17

**Authors:** Hafiz Azhar Ali Khan, Waseem Akram

**Affiliations:** 1 Institute of Agricultural Sciences, University of the Punjab, Lahore, Pakistan; 2 Department of Entomology, University of Agriculture, Faisalabad, Pakistan; Johns Hopkins University, United States of America

## Abstract

**Background:**

Diarrhea is an important cause of childhood mortality in developing countries like Pakistan because of unhygienic conditions, lack of awareness, and unwise use of preventive measures. Mechanical transmission of diarrheal pathogens by house flies, *Musca domestica*, is believed as the most effective route of diarrhea transmission. Although the use of insecticides as a preventive measure is common worldwide for the management of house flies, success of the measure could be compromised by the prevailing environmental temperature since it significantly affects toxicity of insecticides and thus their efficacy. Peaks of the house fly density and diarrheal cases are usually coincided and season specific, yet little is known about the season specific use of insecticides.

**Methodology/Principal Findings:**

To determine the temperature-toxicity relationship in house flies, the effect of post-bioassays temperature (range, 20–34°C) on the toxicity of seven insecticides from organophosphate (chlorpyrifos, profenofos), pyrethroid (cypermethrin, deltamethrin) and new chemical (emamectin benzoate, fipronil, spinosad) classes was evaluated by using a feeding bioassay method. From 20–34°C, the toxicities of chlorpyrifos, profenofos, emamectin and fipronil increased 2.10, 2.93, 2.40 and 3.82 fold (i.e. positive temperature coefficient), respectively. Whereas, the toxicities of cypermethrin, deltamethrin and spinosad decreased 2.21, 2.42 and 3.16 fold (i.e. negative temperature coefficient), respectively.

**Conclusion/Significance:**

These findings suggest that for the reduction in diarrheal cases, house flies should be controlled with insecticides according to the prevailing environmental temperature. Insecticides with a positive temperature coefficient may serve as potential candidates in controlling house flies and diarrhea epidemics in hot season and vice versa.

## Introduction

Diarrheal infections are amongst the major causes of morbidity and mortality among all age groups [1], particularly the under-five age group [2], around the globe. Controlling these infections is an important task to meet the Millennium Development Goal (MDG) no. 4 “*Reduce child mortality: Reduce by two thirds, between 1990 and 2015, the under-five mortality rate*”. Although death rates of the children fall in richer developing regions, the condition is very alarming in the poorest regions like Southern Asia and sub-Saharan Africa. The statistics revealed that, in 2011, both of the regions contributed 5.7 million of the 6.9 million under five deaths worldwide, mostly from preventable diseases [3]. Of these, diarrheal infections are the second leading cause responsible for 1.5 million child deaths each year. In Pakistan, a child under the age of five usually receive on average five episodes of acute watery diarrhea per annum, and it is believed as the leading cause of childhood deaths [4], [5]. Diarrhea is caused by a variety of pathogens in the genera like *Aeromonas*, *Campylobacter*, *Escherichia*, *Salmonella* and *Vibrio* [6]. Although illiteracy, poor water supply, sanitation and hygiene play a significant role in very high diarrhea cases, mechanical transmission of the pathogens by the common house flies, *Musca domestica* L., (Diptera: Muscidae) has been considered the most effective route of diarrheal pathogens transmission [5], [7], [8]. Outbreaks of childhood diarrhea have been assumed as closely related to the seasonal abundance of house flies while their control with insecticides and/or other measures resulted in the decline of such outbreaks [4], [5], [8], [9], and it was suggested that the preventive measures which could interrupt disease transmission by house flies need to be developed and promoted [2], [10]. The severity of the problem heightens the need to curb the menace of diarrheal infections by controlling house flies in an effective manner in order to reduce annual deaths.

In areas of intensive animal/dairy farming, like the Punjab province of Pakistan, animal manure (feces) in the farms is generally thought to aid in the multiplication and spread of house flies in and/or around the farms, and ultimately transmission of different diseases to animals and human beings nearby [11]. Synthetic insecticides from all the classes: organochlorine, organophosphate, carbamate, pyrethroid and new chemicals, have been used to control house flies worldwide. Since preventive measures like the judicious and appropriate use of insecticides could be very beneficial in situations where high fly density is associated with diarrheal epidemics [2], but information regarding the season specific use of insecticides is lacking. A recent dairy farmers’ survey in Punjab, Pakistan [4] revealed that the farmers were practicing intensive animal farming, but had weak knowledge regarding the problems associated with house flies and effective management practices. In addition, the survey revealed that there was a lack of systematic management plan for house flies which ultimately lead to malpractices like inappropriate chemical use with no importance to season specific insecticides, storage of animal manure in open places etc. These measures resulted in the development of insecticide resistance in house flies [12], [13] and may also be responsible for the high rate of diarrhea cases in urban and rural settings in Pakistan.

In the Punjab province of Pakistan, house flies occur throughout the year with fluctuations depending upon different environmental conditions, temperature in particular. Since an insect’s body temperature changes with its surroundings, environmental temperature can compromise disease vector control by influencing the effectiveness or toxicity of the insecticides. High alerts regarding the acute watery diarrhea in Pakistan have been declared by the National Institute of Health (NIH) in collaboration with the World Health Organization (WHO) in summer and winter seasons 2013 [14] and in the previous years. Studies in Pakistan revealed that a high rate of diarrhea cases coincided with the house fly’s peak density seasons between March and June [2], [10]. These are the summer months in Pakistan; however, keeping in view the above stated alerts, winter months should not be ignored. In this scenario, season specific measures are needed which could control house flies and ultimately diarrhea transmission in the community. Insecticides play a crucial role in the management of insect- or vector-borne diseases [15], but metabolic activities in insects, responsible for insecticide degradation, are highly temperature-dependent. Since insects’ body temperature changes with its surroundings, therefore surrounding temperature can compromise disease vector control by influencing the insecticide toxicity. For example, *Aedes albopictus* and *Culex restuans* mosquitoes were more susceptible to malathion at 30°C than at 20°C [16]. This temperature-toxicity relationship can be determined by calculating the temperature coefficient of an insecticide. An insecticide with a positive temperature coefficient becomes more toxic with the increase in temperature, whereas, those with a negative temperature coefficient become more toxic at lower temperatures [17]. Pyrethroid and organophosphate insecticide classes, for example, usually have a negative and positive temperature coefficient, respectively [18]. However, some studies also revealed variation in the toxicity within a given insecticide class [16], [19], between insect species and temperature range tested [16], [20]. Therefore, generalization of the temperature-toxicity trend could be misleading within a given class, and for different insect species. Therefore, by considering the temperature-toxicity relationship, malpractices (stated above), seasonal coincided peaks in diarrhea and house flies, and the importance of insecticidal control of flies, information regarding the season specific insecticides or temperature-toxicity relationship in house flies should be generated which could be helpful to health professionals, entomologists, policy makers and livestock owners, and for the people living in and/or around potential fly breeding areas. Such studies are rare in the field of house fly-borne disease management by improving the existing preventive measures, particularly in Pakistan. Present study focus on to explore the correlation between temperature and the toxicity of insecticides from different classes by calculating the temperature coefficient. It is expected that the result could be helpful in selecting appropriate insecticides with positive temperature coefficients in summer and negative temperature coefficients in the winter months of the year, and the ultimate effect on the reduction in diarrheal infections.

## Materials and Methods

### Insects

Adult house flies of both sexes were collected from a dairy farm in Lahore, Punjab, and brought to the laboratory. The flies were reared by following the methodology of Khan et al. [21], [22] for three generations without exposure with insecticides before insecticidal bioassays. The flies were placed in screened mesh cages (40×30×30 cm) and provided powdered milk and sugar (1∶1), and water. In addition, for egg laying and immature development, a medium containing powdered milk, sugar, yeast, grass meal and wheat bran at a ratio of 0.3∶0.3∶1∶2∶4 by weight, respectively, was also provided. The flies were reared under the standard laboratory conditions: 25±2°C, 60±5% RH and 12∶12 light: dark photoperiod. No specific permit was required to collect house fly samples from the dairy farm as it was privately owned and collection was made merely by arrangement with the owner. Since the house fly is not an endangered species, no permission was required from any concerned authority in Punjab, Pakistan [21].

### Insecticides and Bioassays

Commercial formulations of seven insecticides from different classes: two organophosphate [chlorpyrifos (Lorsban 40 EC), profenofos (Curacron 50 EC, Syngenta)], two pyrethroid [cypermethrin (Arrivo 10 EC, FMC); deltamethrin (Decis Super 10.5 EC, Bayer Crop Science)], and three new chemical insecticides [emamectin benzoate (Proclaim 019EC, Syngenta); fipronil (Regent 36EC, Bayer Crop Sciences); spinosad (Tracer 24SC, Dow Agro Sciences)], were selected for testing the effect of temperature (20, 27, and 34°C ) on toxicity.

At least five concentrations (causing >0 and <100% mortality) as serial dilutions of each insecticide were made in distilled water+sugar solution (20%) and tested at each temperature. All the insecticide concentrations were made on the day of experiment and replicated thrice. Feeding bioassays were done by following an approved method (#0026 available at http://emethods.irac-online.org/) by the Insecticide Resistance Action Committee (IRAC). In short, twenty 3–5-day-old female flies were introduced into plastic containers (250 ml) and provided two pieces of cotton dental wick (2 cm length) moistened with a sugar water solution containing different concentrations of insecticides. In control plastic containers, cotton wicks soaked in 20% sugar solution without toxicant were provided to flies. The containers were immediately placed in growth chambers set at 20±1, 27±1, or 34±1°C and 60±5% RH with a 12∶12 (L/D) h photoperiod. The house flies mortality counts were made at 48 h of post-exposure to insecticides and all the ataxic flies were assumed dead [22].

### Data Analysis

Mortality data of three replicates against each insecticide concentration were pooled and analyzed by Probit analysis using the software SPSS (Version 10.0 for windows, SPSS Inc., Chicago, the USA) to determine median lethal concentrations (LC_50_). The toxicity of insecticides was considered significantly different if the confidence intervals (CIs) at LC_50_ level did not overlap [23].Temperature coefficients of each insecticide tested at different temperatures were calculated as the ratio of higher to the lower LC_50_ and called negative when the lower LC_50_ was at a lower temperature and positive when the lower LC_50_ at a higher temperature [18].

## Results

The toxicity of profenofos and chlorpyrifos was found positively correlated with the temperature range tested. Based on LC_50_ values, the toxicity of profenofos increased significantly to 1.53 and 1.91 folds at temperatures 27 and 34°C, when compared with the toxicity at 20°C (non-overlapping of 95% CIs; Table 1). Similarly for chlorpyrifos, the toxicity was increased 1.43 and 1.47 fold at 27 and 34°C respectively, when compared with the toxicity at 20°C. Both the products showed overall positive temperature coefficients 2.93 and 2.10 fold, respectively, for the temperature range tested.

**Table 1:** Effect of temperature on insecticide toxicity to the adult female *Musca domestica.*

Insecticide	Temp. (°C)	*n* a	LC_50_ b (95% CI)(µg/ml)	Fit of probit line			Temp. Coefficient^c^	
				Slope	?^2^ (df)	P	7°C	14°C
Chlorpyrifos	20	360	11.19 (9.36–13.58)	2.12±0.22	0.45 (3)	0.93		
	27	420	7.80 (6.45–9.54)	1.83±0.17	0.14 (4)	0.99	1.43	
	34	480	5.32 (4.45–6.39)	1.93±0.16	3.87 (5)	0.56	1.47	2.10
Profenofos	20	420	30.38 (25.12–37.98)	2.06±0.21	2.24 (4)	0.69		
	27	420	19.82 (16.54–23.18)	2.00±0.19	4.83 (4)	0.31	1.53	
	34	480	10.37 (8.58–12.61)	1.76±0.14	4.72 (5)	0.45	1.91	2.93
Cypermethrin	20	480	208.31 (174.30–250.7)	1.95±0.15	7.41 (5)	0.19		
	27	480	303.56 (255.12–366.55)	2.10±0.81	5.62 (5)	0.34	-1.46	
	34	420	461.57 (383.67–572.22)	2.11±0.21	2.74 (4)	0.60	-1.52	-2.21
Deltamethrin	20	480	48.44 (39.28–59.23)	1.61±0.13	4.79 (5)	0.44		
	27	480	78.58 (65.24–94.92)	1.82±0.15	4.31 (5)	0.51	-1.62	
	34	420	117.28 (97.51–141.99)	1.91±0.17	6.63 (4)	0.15	-1.49	-2.42
Emamectin	20	420	87.56 (72.68–107.66)	1.95±0.19	1.38 (4)	0.84		
	27	540	47.72 (38.77–59.51)	1.51±0.12	7.90 (6)	0.24	1.83	
	34	480	36.50 (30.06–45.14)	1.77±0.16	4.88 (5)	0.43	1.31	2.40
Spinosad	20	420	1.48 (1.25–1.75)	2.24±0.19	5.40 (4)	0.25		
	27	420	2.56 (2.12–3.17)	1.86±0.18	2.65 (4)	0.61	-1.73	
	34	360	4.67 (3.87–5.87)	2.26±0.26	2.37 (3)	0.50	-1.82	-3.16
Fipronil	20	360	53.87 (42.93–74.10)	2.15±0.28	0.89 (3)	0.82		
	27	420	26.96 (22.41–33.31)	2.06±0.20	0.81 (4)	0.93	2.00	
	34	420	14.12 (10.28–19.84)	2.38±0.19	7.03 (4)	0.14	1.91	3.82

a = number of house flies tested.

b = median lethal concentration.

c = Ratio of higher to lower LC50 value for 7 and 14°C differences in temperature. A negative coefficient indicates a higher LC_50_ at the higher temperature [18].

In contrast to organophosphates, pyrethroids showed a negative association with temperatures tested. The toxicity of cypermethrin decreased by 1.46 and 1.52 fold at 27 and 34°C respectively, when compared with the toxicity at 20°C (non-overlapping of 95% CIs; Table 1), with an overall −2.21 temperature coefficient. Similarly, the toxicity of deltamethrin decreased with the increase in temperature range, with an overall negative temperature coefficient of −2.42.

In case of new chemical insecticide formulations, emamectin benzoate and fipronil showed positive association at all the temperatures, whereas spinosad had a negative association. The toxicities of emamectin and fipronil increased by 1.83 and 2 folds at the temperature 27°C respectively, and 1.31 and 1.91 folds at 34°C respectively, when compared with the toxicities at 20°C (non-overlapping of 95% CIs; Table 1). However, the toxicity of spinosad decreased with increase in temperature with an overall negative temperature coefficient of 3.16.

## Discussion

In the present study the influence of three different temperature levels was determined on the toxicity of different insecticides in house flies. Management of this notorious pest is very important for minimizing deadly disease transmissions (e.g. cholera, diarrhea, dysentery, poliomyelitis, viral hepatitis A & E) both in animals and human beings, and chemical control is amongst the major controlling measures [22]. Chemicals used against house flies usually caused mortality by disrupting the functions of the nervous system [21]. Different metabolic activities in insects’ body, responsible for the degradation of insecticides and normal functioning of the nervous system, are highly temperature dependent [24]. Since an insect’s body temperature changes with its surroundings, environmental temperature can compromise disease vector control by influencing the effectiveness or toxicity of the insecticide [17]. Both of the organophosphates tested in the present study showed temperature dependent toxicities, with chlorpyrifos being more toxic than profenofos at the highest temperature range (34°C) tested. Organophosphate insecticides usually have a positive association with surrounding temperatures, therefore, these could be assumed theoretically to perform well in high temperature conditions [17].The results are in accordance with those reported on organophosphates but with different insect pests [20], [25], [26]. A probable reason for this increased toxicity could be increased penetration of the organophosphates into the body of house flies. At low temperatures, the toxicity of organophosphate insecticides decrease due to the decrease in a biological process called biotransformation [27]. A lot of enzymatic activities are responsible for different chemical changes in the compound during biotransformation. The decrease in biotransformation results in elevated level of the original compounds which are less toxic than the compounds formed through biotransformation. Similar to organophosphates, emamectin and fipronil also showed positive association with fipronil being more toxic at the highest temperature tested. Therefore, in theory, the above insecticides should perform better under warmer climates.

Among pyrethroids, cypermethrin had high LC_50_ values as compared to those of deltamethrin. This could be due to the fact that cypermethrin was being heavily used via dipping method to treat animals at the dairy farm which might caused selection pressure on house flies [12]. In contrast with organophosphates, pyrethroids tested in the present study showed a negative association with temperature. The results are in accordance with already reported on the impact of temperature on pyrethroid toxicity with different insect species [18], [26], [28]. There could be many reasons for the decreased toxicity of pyrethroids at higher temperatures as have been explored by different researchers [27], [29], [30]. Being axonic poisons, pyrethroids control the movement of sodium ions during the movement of nerve impulse. The sensitivity of neurons increases between the temperatures 15 to 20°C, which results in repetitive nerve firing. But the reverse has been observed at higher temperatures 30–35°C [30]. At low temperatures, pyrethroid-exposed neurons receive a high concentration of the toxicant because of reduced biotransformation, and are more sensitive to the resulting stimulus due to a prolonged duration of steady-state resting potential [27]. The decrease in biotransformation results in the accumulation of the original compound which, in contrast to organophosphates, are more toxic than the compounds created in the process of biotransformation. In addition, sodium influx increases due to the stability of open sodium channels at low temperatures [27], [31]. Resultantly, pyrethroid toxicity in house flies increased at low temperatures, perhaps due to any one and/or combination of the reasons stated above. Similar to pyrethroids, spinosad also showed a negative association with temperatures tested. Our results on the toxicity of spinosad are in agreement with bioassays on *O. nubilalis* [18], where an inverse relationship between the toxicity and temperature was observed. Temperature is an important factor in affecting the toxicity of microbial insecticides, since spinosad is a microbial insecticide [32] which might be a probable reason for the decreased toxicity at higher temperatures. However, further research is needed to completely understand the phenomenon of decreased toxicity of spinosad with the increase in temperature. Theoretically, keeping in view the inverse relationship between temperature and toxicities of the pyrethroids and spinosad, these insecticides should perform better at low temperatures in the field.

Assessing the impact of temperature on the toxicity of different insecticides against a target insect pest is critical in implementing chemical-based management strategies with reference to environmental conditions in a given locality [20]. House flies are cosmopolitan pests with the ability to survive in a wide range of climates [11]. Since regional dairy farms have abundant quantities of animal manure, and therefore likely, unhygienic conditions, they have been assumed as major breeding and expansion sources of house flies and might be a source of diarrhea and other diseases’ transmission in nearby communities [4]. In addition, unplanned and inappropriate use of insecticides could increase the possibility of different disease epidemics. In this scenario, there was a need to have temperature specific insecticides for better management of house flies. As stated above, house flies occur throughout the year with fluctuations depending upon the different environmental conditions, including temperature. Outbreaks of intestinal diseases like diarrhea in urban and rural settlements are closely related to the seasonal abundance of house flies while their control resulted in the decline of such outbreaks [4]. Keeping in view the alarming situation of diarrhea in summer and winter season in Pakistan, appropriate management plans were needed to prevent disease epidemics by improving preventive measures planned for house flies. This could be achieved by selecting appropriate insecticides according to the seasons. Based on our results, organophosphate (chlorpyrifos, profenofos), emamectin and fipronil should be potential candidates for controlling house flies during the hot summer months in Punjab. The average temperature in June in the plains including Punjab has been reported to be 38°C [33] and March-June was already reported as the peak house fly density and diarrheal cases period. In contrast, winters are cold; the diurnal variation in temperature may be as much as 11–17°C. Although summer is the expected peak season of both flies and diarrhea, winter also demands the house flies control to prevent large population outbreaks in summer and ultimately diarrheal infections. Based on the results, pyrethroid (cypermethrin and deltamethrin) and spinosad could provide effective control of house flies in winters. Hence, large outbreaks of house fly populations and diarrheal epidemics could be controlled by using appropriate insecticides according to the prevailing environment conditions. Moreover, rotation of insecticides in summer and winter will also reduce selection pressure on house flies and ultimately delay the development of insecticide resistance [34].

In conclusion, toxicity of chlorpyrifos, profenofos, emamectin and fipronil to house flies revealed a direct relationship with the temperature range tested. Whereas, an inverse relationship between temperature and toxicity of cypermethrin, deltamethrin and spinosad was observed. The results could be helpful in designing effective chemical-based management plans for house flies in summer and winter seasons. Owing to poverty, lack of awareness and education, people of the developing countries like Pakistan usually don’t consider house flies as a major pest. Resultantly, a major share of their hand to mouth earnings is invested every year in the medication of fly-borne diseases rather than adopting simple preventive measures against house flies (Khan and Akram, personal communication). As recommended earlier [4], training of the livestock owners and the general public regarding the problems associated with house flies, and effective management of animal manure and human feces could reduce house flies densities and their capability to transmit disease pathogens. Such training programs should be coupled with the information of season or time specific insecticide use. Considering the study’s constraints of time and cost, we were not able to provide a long term field analysis of the use of specific insecticides against house flies and the ultimate impact on diarrheal cases. Despite this limitation, the findings of the present study have important implications for the management of house flies and ultimately childhood deaths by diarrheal infections.
